# Supplementary material for: Bioguided Fractionation of Local Plants against Matrix Metalloproteinase9 and Its Cytotoxicity against Breast Cancer Cell Models: In Silico and In Vitro Study
Source: Molecules. 2020 Oct 14;25(20):4691. doi: 10.3390/molecules25204691 (PMC7587335; doi:10.3390/molecules25204691)
Supplement: Supplementary file 1 [file molecules-25-04691-s001.pdf]

molecules

MDPI

Supplementary Materials

Bioguided Fractionation of Local Plants against Matrix Metalloproteinase9 and Its Cytotoxicity against Breast Cancer Cell Models: *In Silico* and *In Vitro* Study

Maywan Hariono[1]*, Rollando Rollando[2], Jasson Karamoy[1], Pandu Hariyono[1], M. Atmono[1], Maria Djohan[1], Wiwy Wiwy[1], Rina Nuwarda[3], Christopher Kurniawan[2], Nurul Salin[4], and Habibah Wahab[5]

1. Drug Discovery Research Group, Faculty of Pharmacy, Sanata Dharma University, Campus III, Paingan, Maguwoharjo, Depok, Sleman 55282, Yogyakarta, Indonesia; mhariono@usd.ac.id
2. Pharmacy Program, Faculty of Science and Technology, Ma Chung University, Malang 65151, Indonesia; ro.llando@machung.ac.id
3. Faculty of Pharmacy, Padjadjaran University, Jatinangor, Sumedang 45363, West Java, Indonesia; rina.nuwarda@unpad.ac.id
4. Malaysian Institute of Pharmaceuticals and Nutraceuticals, National Institute of Biotechnology Malaysia, Halaman Bukit Gambir, 11900 Bayan Lepas, Pulau Pinang, Malaysia; hanim@nibm.my
5. Pharmaceutical Technology Department, School of Pharmaceutical Sciences and USM-RIKEN Centre for Ageing Science (URICAS), Universiti Sains Malaysia, 11800 Minden, Pulau Pinang, Malaysia; habibahw@usm.my

* Correspondence: mhariono@usd.ac.id ; Tel.: +62-895-0628-6901

Table S1: The binding affinity of published PEX9 inhibitors predicted by molecular docking compared to their experimental dissociation constant upon PEX9 inhibition.

Ligand	Free Energy of Binding (kcal/mol)	K_d[18, 19] (μM)	Activity Classification
alford_3d	-8.70	0.49	TP
alford_4d	-8.60	0.31	TP
dufour_2	-7.90	2.00	FP
alford_4c	-7.70	0.65	TP
alford_3a	-7.60	0.56	TP
alford_1d	-7.60	1.33	uncertain
alford_3b	-7.50	0.49	TP
alford_4a	-7.40	0.42	TP
alford_1c	-7.00	4.22	FP
alford_1a	-7.00	1.33	uncertain
alford_2c	-6.80	0.87	TP
alford_2a	-6.70	1.15	uncertain
alford_3c	-6.70	0.32	TP
alford_4b	-6.60	0.87	TP
alford_1b	-6.50	4.87	FP
alford_1f	-5.90	0.49	TP
alford_4e	-5.70	0.87	TP

Uncertain = moderately active

Table S2: The yield of Ageratum conyzoides and Ixora coccinea fractions of ethylacetate and *n*-hexane, respectively.

Plants	Yield (%)			
	**Fraction 1**	**Fraction 2**	**Fraction 3**	**Fraction 4**
Ageratum conyzoides	8.8	15.8	7.0	7.0
Ixora coccinea	6.8	0.3	0.9	NA

Figure S1. The mass spectra of three peaks identified from GC chromatogram of Ixora fraction 1. The three GC chromatogram peaks with Rt 10.709 min, 12.380 min, and 14.153 min are detected as compounds having mass/ ion: 548 (base peak 355), 529 (base peak 73), and 528 (base peak 73), respectively.The base peak informs the most stable fragment during electron impact in MS characterization.

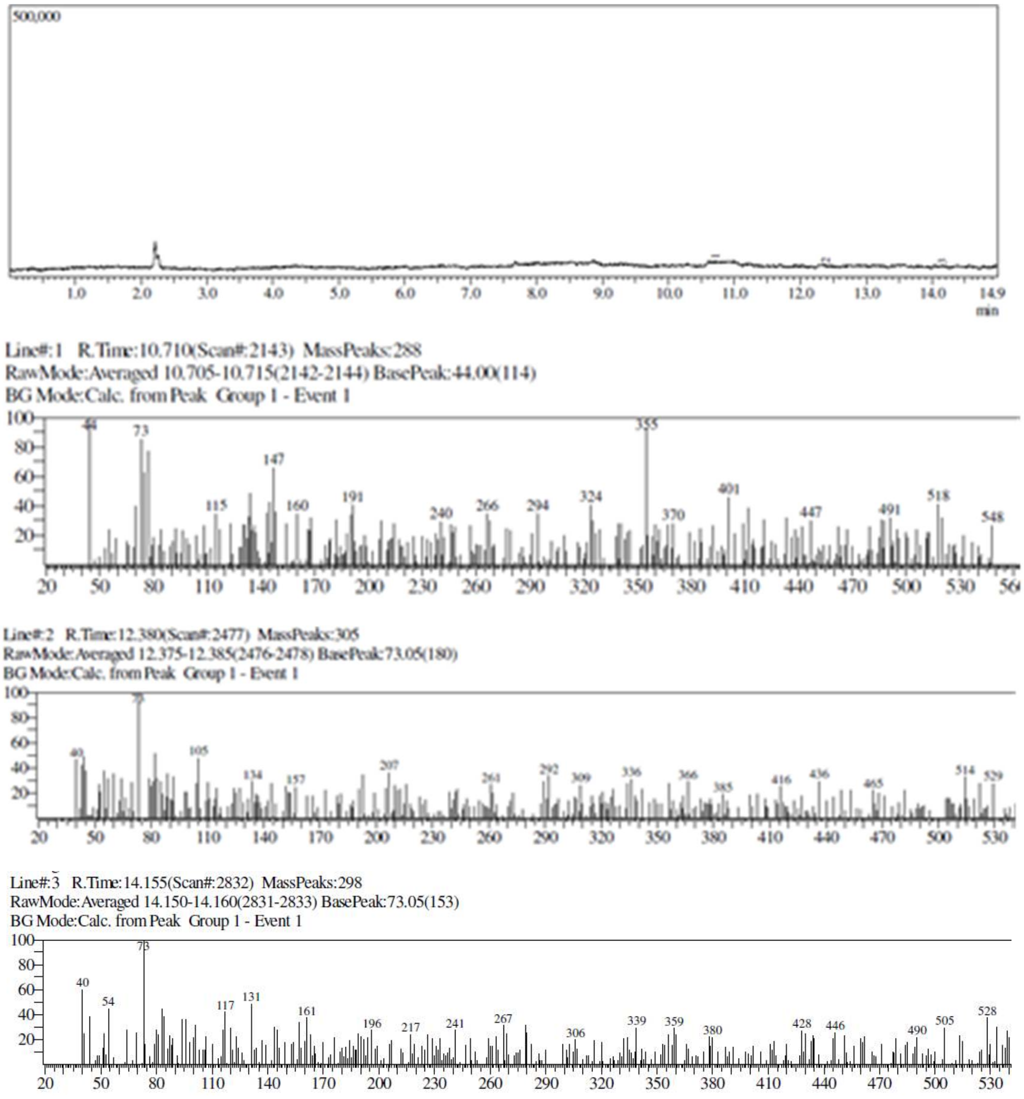

© 2020 by the authors. Submitted for possible open access publication under the terms and conditions of the Creative Commons Attribution (CC BY) license (http://creativecommons.org/licenses/by/4.0/).
